# A Mini-Review: Recent Advances in Coumarin-Metal Complexes With Biological Properties

**DOI:** 10.3389/fchem.2021.781779

**Published:** 2021-12-01

**Authors:** Łukasz Balewski, Sylwia Szulta, Aleksandra Jalińska, Anita Kornicka

**Affiliations:** Department of Chemical Technology of Drugs, Faculty of Pharmacy, Medical University of Gdańsk, Gdańsk, Poland

**Keywords:** coumarin, 2*H*-chromen-2-one, metal complexes, biological activity, fluorescent properties

## Abstract

The coumarin nucleus is a recurring motif in both natural and synthetic compounds that exhibit a broad spectrum of biological properties including anticoagulant, anti-inflammatory, antioxidant, antiviral, antimicrobial and anticancer agents as well as enzyme inhibitors. On the other hand, it has been reported that the incorporation of a metal ion into coumarin derivatives can increase the activity of such complexes compared to coumarin-based ligands. Accordingly, some of them have been found to display promising antioxidant, antitumor or antibacterial activities. This mini-review briefly summarizes the recent development of coumarin-metal complexes with proven biological properties. The attention is also paid to agents for which practical applications in the detection of biologically important species may be found.

## Introduction

Coumarins belong to a family of large and extensively studied compounds containing 2*H*-1-benzopyran-2-one core structure, which consists of fused benzene and *α*-pyrone rings. This heterocyclic system is also known as 1,2-benzopyrone, 2*H*-chromen-2-one, 2-oxo-1,2-benzopyran or *o*-hydroxycinnamic acid lactone. The history of coumarins can be traced back to 1820 when H. A. Vogel first isolated the simplest member of this family - coumarin from the *tonka beans*. Later on, this compound was first synthesized by W. M. Perkin in 1868. Both natural and synthetic coumarins are endowed with a great therapeutic potential due to the wide spectrum of biological properties including anticancer, antimicrobial, antiviral, anti-inflammatory, neuroprotective, and antioxidant activities. Hence, the coumarin skeleton can be foresighted as a privileged scaffold for the design and synthesis of pharmacologically active compounds (Pereira et al., 2018; Srikrishna et al., 2018; Stefanachi et al., 2018; Akkol et al., 2020; Al-Warhi et al., 2020; Garg et al., 2020; Mishra et al., 2020; Patel and Banerjee., 2020).

Additionally, physicochemical properties and biological activities of coumarins might be enhanced by combining coumarin moiety with other chemical species such as, for example, metal ions. The literature survey reveals that several properties of the organometallic complexes offer great opportunities in the development of new compounds with specific and new modes of action. In fact, incorporation of metals such as cobalt, copper, zinc, silver, platinum, palladium, or iridium, into ligand molecules with biological activity has been implemented in the development of novel coumarin-based complexes with better pharmacological activity. As a consequence, a large number of coumarin-based metal complexes have been synthesized in order to obtain more potent molecules (Gasser Metzler-Nolte N., 2012; Noffke et al., 2012; Ibrahim et al., 2019; Parveen et al., 2019; Boros et al., 2020).

**FIGURE 1:**
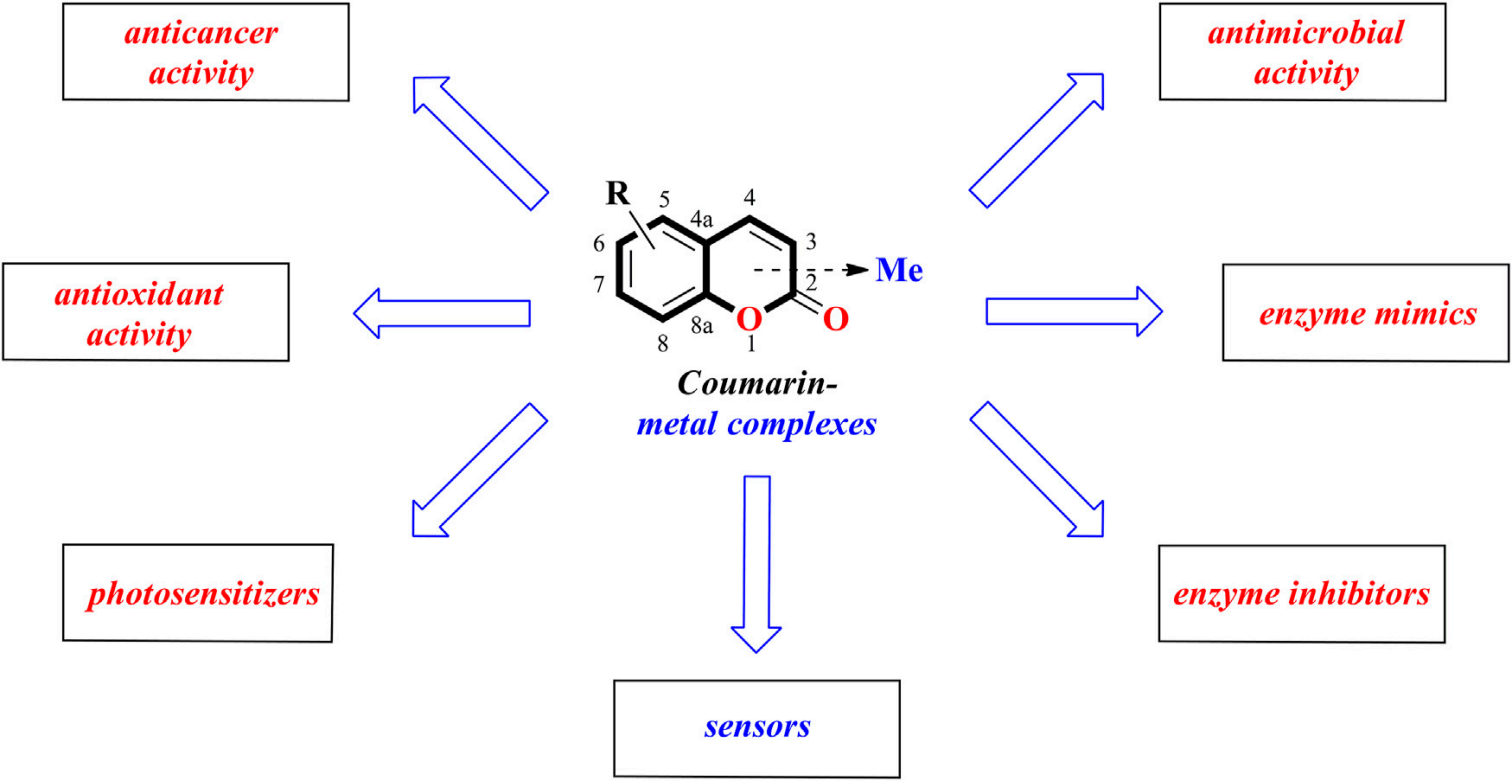
Biological properties of coumarin-metal complexes.

To stay in line with the earlier described review works this mini-review is aimed at summarizing the recent advances in coumarin-metal complexes with anticancer, antibacterial, antioxidant, enzyme-mimicking, or enzyme-inhibiting activities as well as fluorescent properties during the period 2019-2021 (Figure 1) (Annunziata et al., 2020; Balcıoğlu et al., 2020; Song et al., 2020; Carneiro et al., 2021).

## Anticancer Complexes

Although there are many anticancer agents in medical practice, their use is associated with severe side effects and low efficacy. Therefore, the development of novel chemotherapeutics still remains an important area of antitumor drug design. An interesting therapeutically useful anticancer strategy consists in inhibiting cyclooxygenase (COX). On account of the crucial role of COX in cancer-associated inflammation, leading to the development and metastasis of malignancies, several COX-targeted inhibitors may serve as potential anticancer drugs.

In 2019, a series of bi-functional platinum (IV) complexes **1-4** (Table 1) with 7-hydroxycoumarin ligands in axial position were synthesized by Wang and collaborators and evaluated for their antitumor activity (Wang et al., 2019). Promising results were obtained for complex **3** which inhibits the rhCOX-2 activity from 20.1 to 65.8% in a dose-dependent manner. By releasing the appropriate derivative of coumaric acid, complex **3** reduces tumor-associated inflammation. Moreover, it was suggested that in cancers tissues the platinum (IV) complexes **1-4** may be reduced to an equivalent amount of platinum (II) compounds which exert DNA damage showing the bi-functional mechanism of action.

**TABLE 1:** Coumarin-based metal complexes with diverse biological activities.

Chemical structure	Number of complex	Target/Activity	References
**Anticancer complexes**			
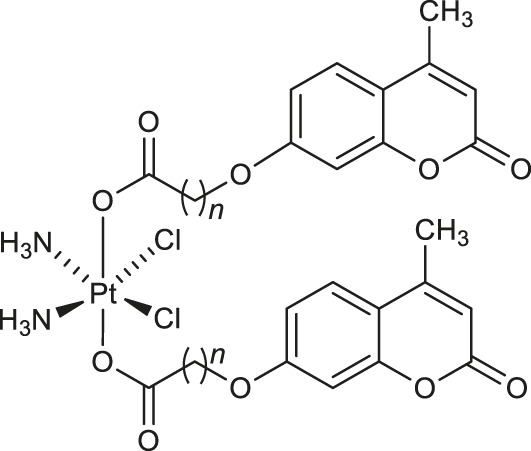	**1**: *n* = 1	rhCOX-2	Wang,et al. (2019)
	**2**: *n* = 3	inhibitors	
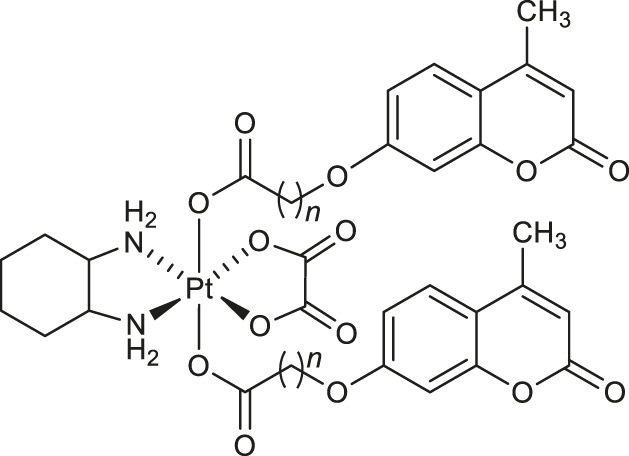	**3**: *n* = 1	rhCOX-2	Wang,et al. (2019)
	**4**: *n* = 3	inhibitors	
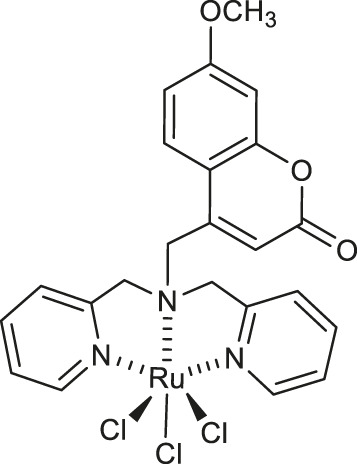	**5**	cervical cancer cell line (HeLa)	Gramni et al. (2019)
		
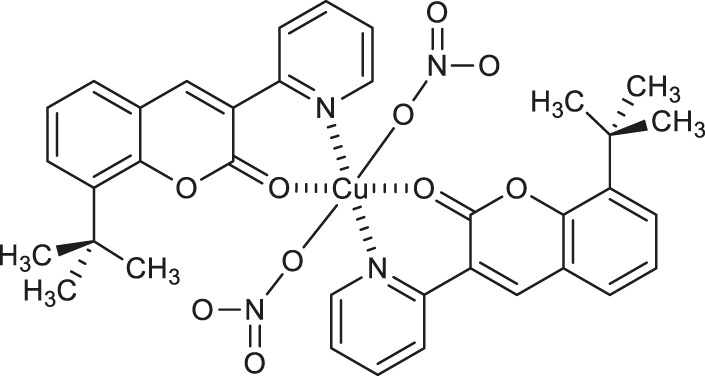	**6**	cervical cancer cell line (HeLa)	Lu et al. (2020)
		
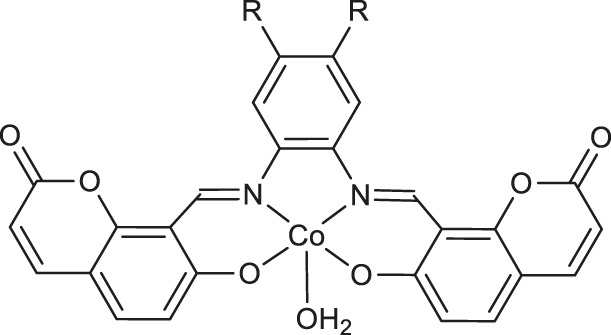	**7**: R = H	cervical cancer cell line (HeLa),generation of ROS	Mestizo et al. (2021)
	**8**: R = OCH_3_		
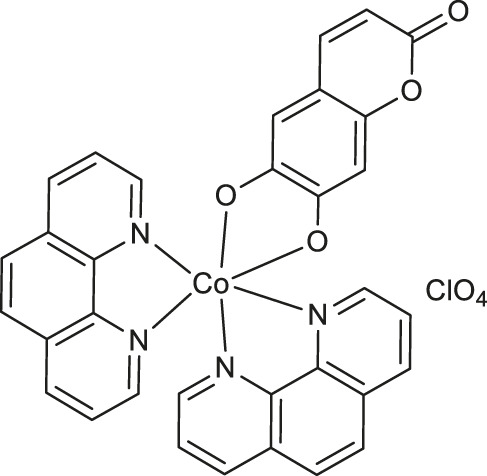	**9**	cervical cancer cell line (HeLa) and human breast cancer cell line (MCF-7)	Sarkar et al. (2021)
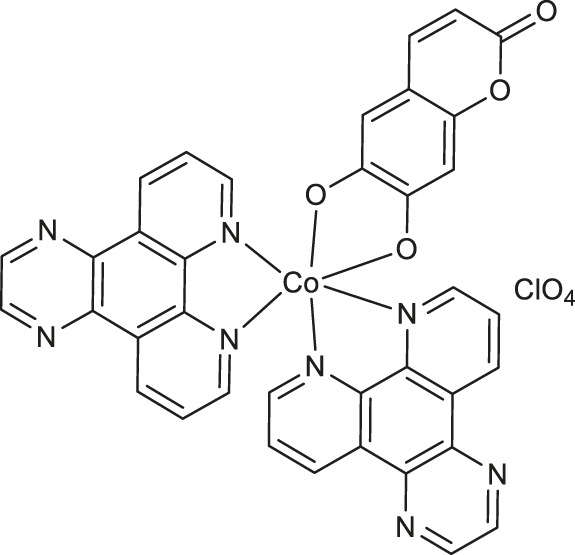	**10**	cervical cancer cell line (HeLa) and human breast cancer cell line (MCF-7)	Sarkar et al. (2021)
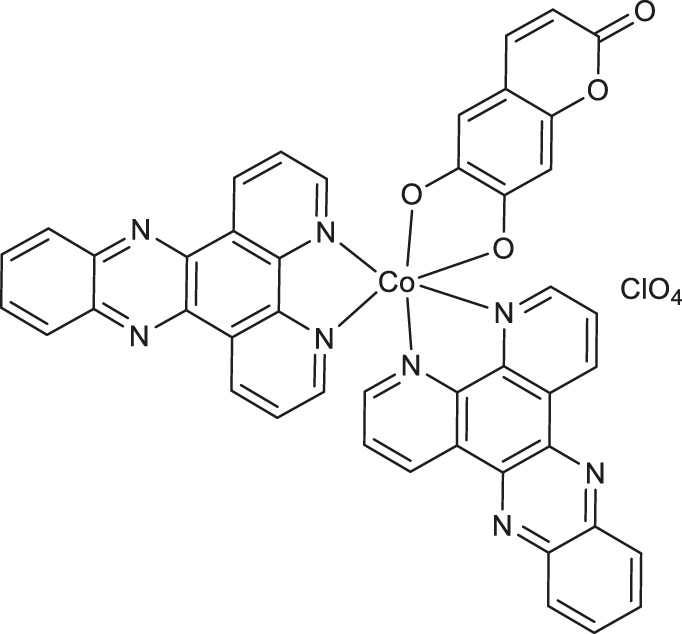	**11**	cervical cancer cell line (HeLa) and human breast cancer cell line (MCF-7)	Sarkar et al. (2021)
**Antibacterial complexes**			
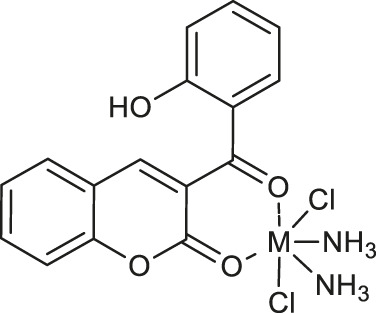	**12**: M = Cu	*Staphylococcus aureus* (ATCC 25923)	Belkhir-Talbi et al. (2019)
	**13**: M = Zn		
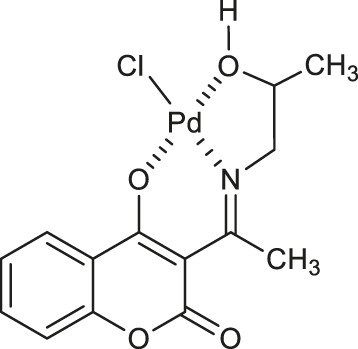	**14**	*B. animalis subsp. lactis, P. aeruginosa, E. coli*	Avdović et al. (2019)
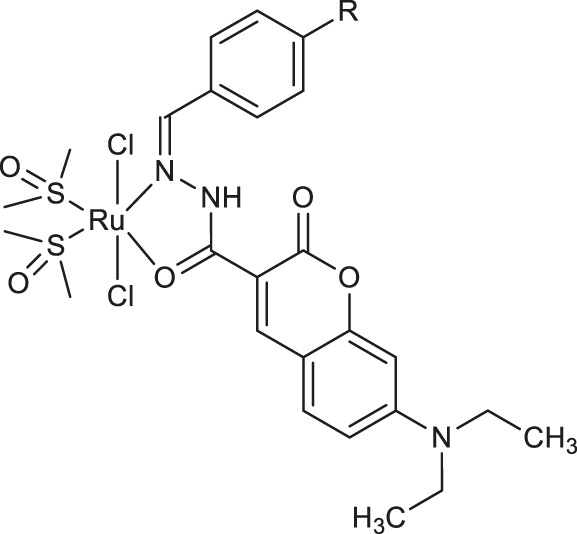	**15**: R = H	*Staphylococcus aureus*	De Almeida et al. (2019)
	**16**: R = Cl		
	**17**: R = Br		
	**18**: R = OCH_3_		
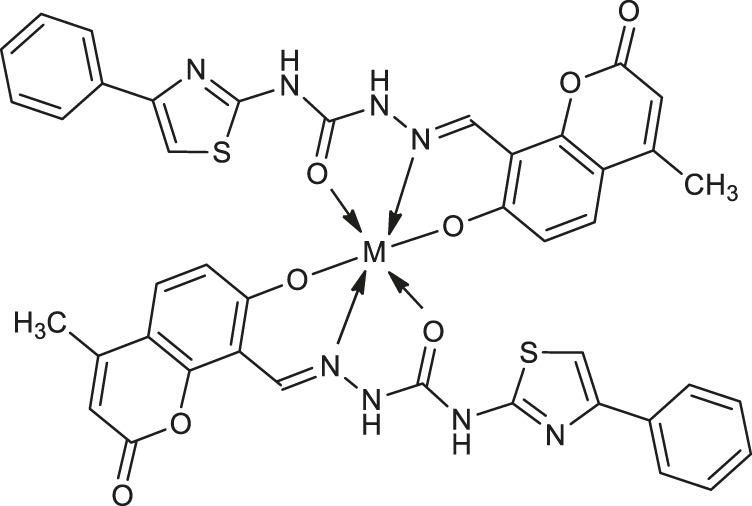	**19**: M = Co	*Bacillus subtilis, Staphylococcus aureus, Salmonella typhi*	Yernale et al. (2020)
	**20**: M = Ni		
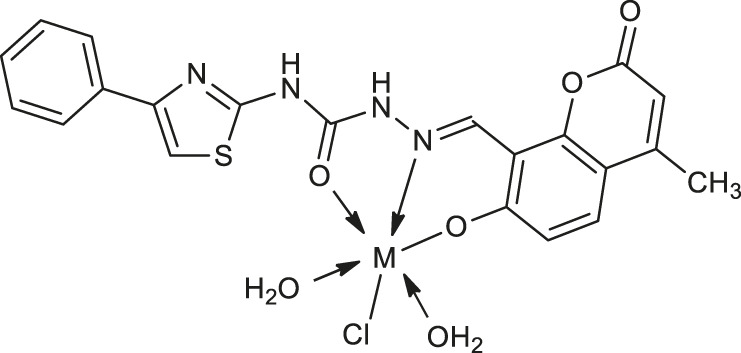	**21**: M = Cu	*Bacillus subtilis*	Yernale et al. (2020)
	**22**: M = Zn	*Staphylococcus aureus*	
		*Salmonella typhi*	
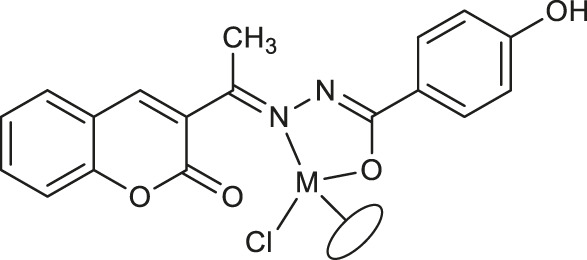	**23**: M = Ir	*Staphylococcus aureus and Bacillus thuringiensis*	Nongpiur et al. (2021)
	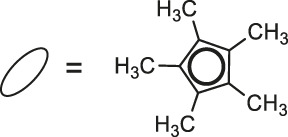		
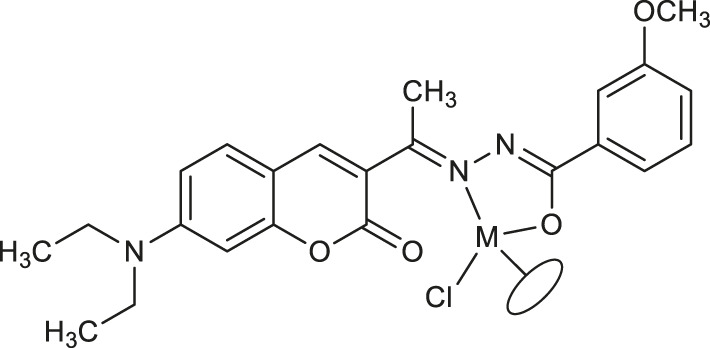	**24**: M = Ru	*Staphylococcus aureus and Bacillus thuringiensis*	Nongpiur et al. (2021)
	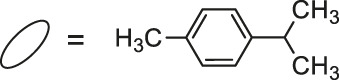		
	**25**: M = Rh		
	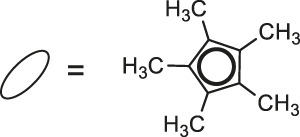		
	**26**: R = Ir		
	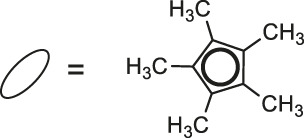		
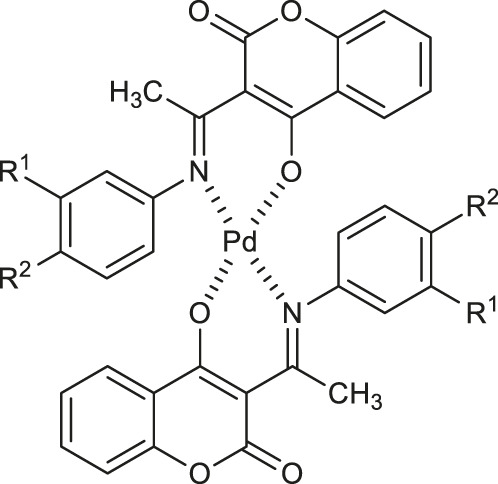	**27**: R^1^ = H; R^2^ = H	SARS-CoV-2 main protease	Milenković et al. (2020)
	**28**: R^1^ = Cl; R^2^ = H		
	**29**: R^1^ = H; R^2^ = Cl		
**Antioxidant complexes**			
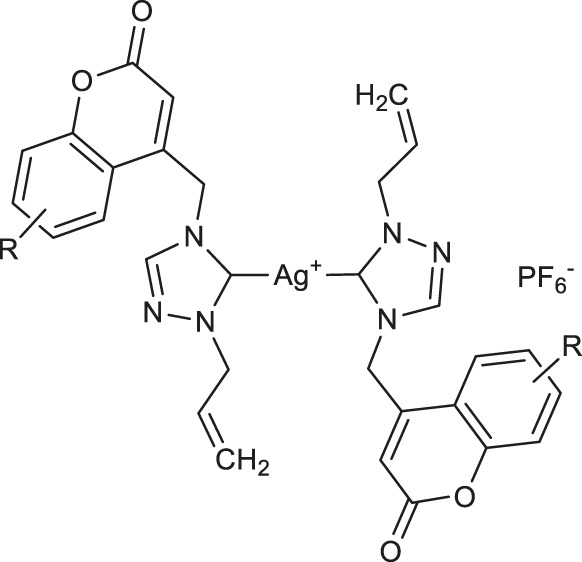	**30**: R = 6-CH_3_	DPPH-based radical scavenging activity	Geetha and Narayana et al. (2020)
	**32**: R = 6-Cl		
	**33**: R = 7,8-benzo		
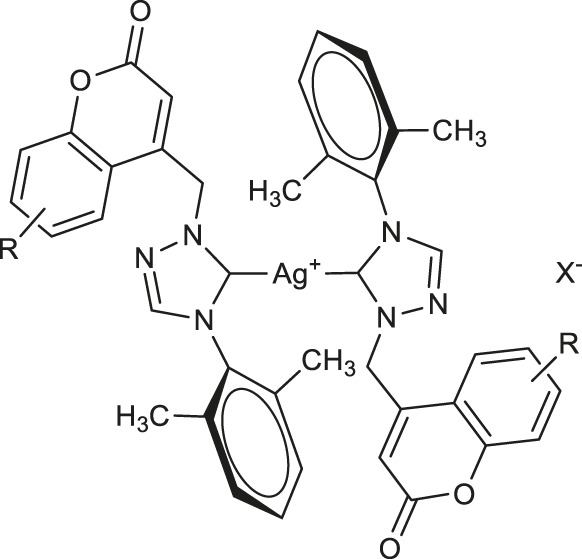	**33**: R = 6-CH_3_	antioxidant activity	Geetha and Małecki. (2020)
	X^−^ = CH_3_COO^−^		
	**34**: R = 7,8-benzo		
	X = Br^−^		
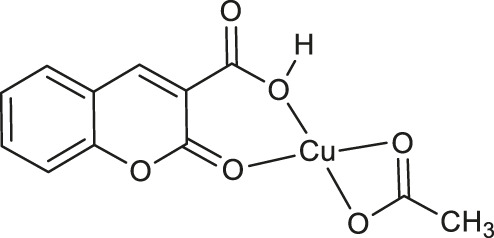	**35**	radical scavenging activity	Souza et al. (2021)
		
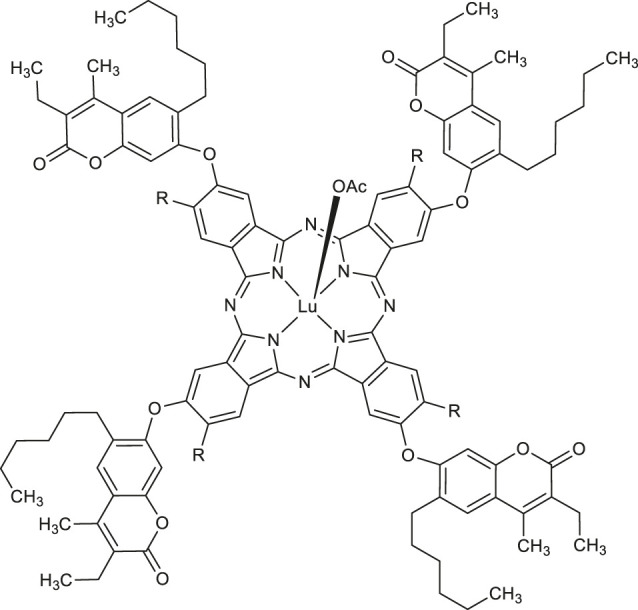	**36**: R = Cl	radical cation scavenging activity	Özdemir and Kӧksoy. (2020)
	**37**: R =		
	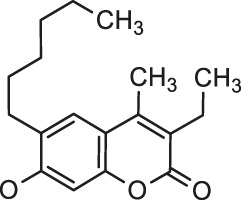		
**Complexes as photosensitizers in photodynamic therapy**			
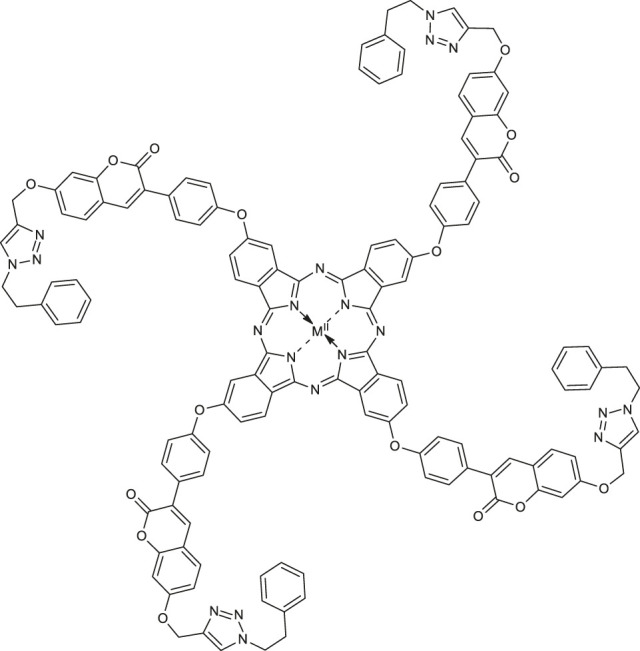	**38**: M^II^ = Zn	type II photosensitizers in photodynamic therapy	Özdemir and Abliatipova. (2020)
	**39**: M^II^ = Mg		
	(peripheral)		
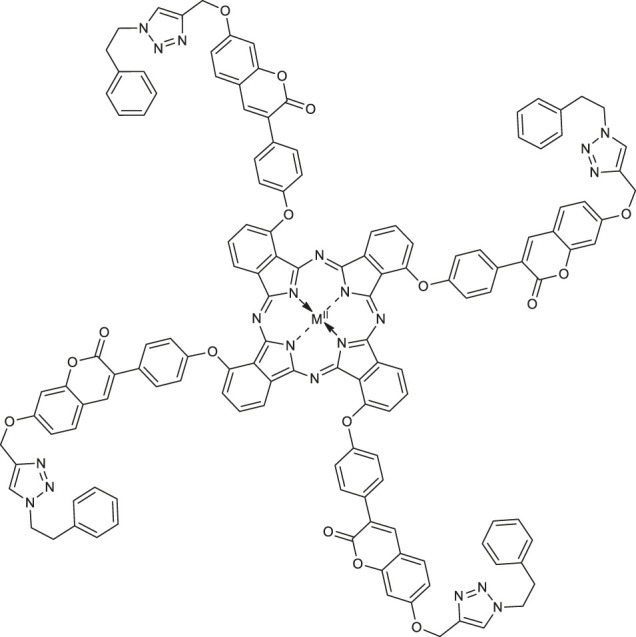	**40**: M^II^ = Zn	type II photosensitizers in photodynamic therapy	Özdemir and Abliatipova. (2020)
	**41**: M^II^ = Mg		
	(non-peripheral)		
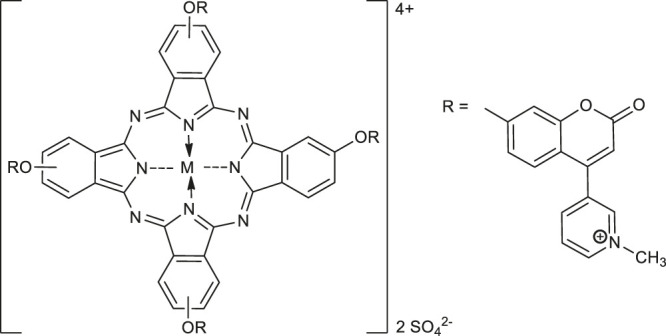	**42**: M = Zn	photosensitizers in photodynamic therapy	Boyar Çamur. (2019)
	**43**: M = InCl		
	**44**: M = Mg		
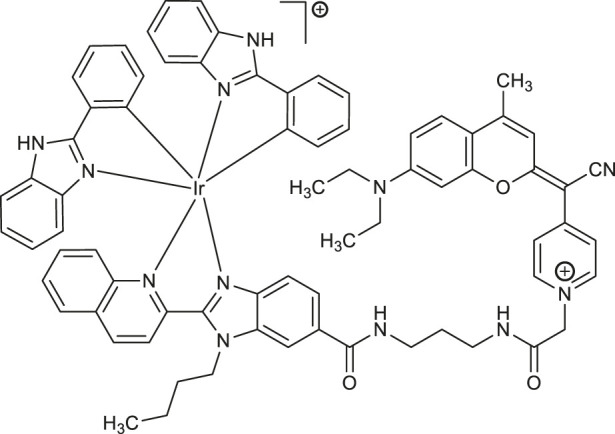	**48**	photosensitizer in photodynamic therapy of cancer: cervical cancer cells (HeLa), prostatic cancer stem cells DU145	Novohradsky and Rovira. 2019
	Ir(III)-COUPY-conjugate		Novohradsky and Markova. (2021)
**Enzyme-mimicking complexes**			
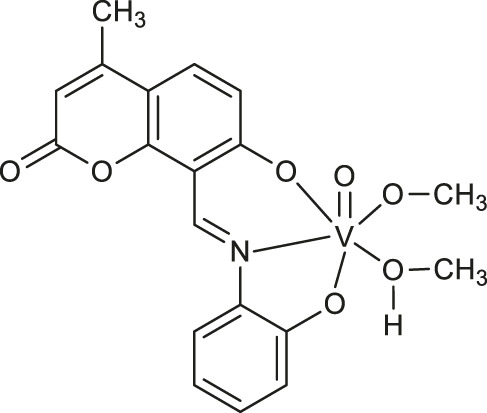	**49**	halo-peroxidase mimicking properties	Majumder and Rajak. (2020)
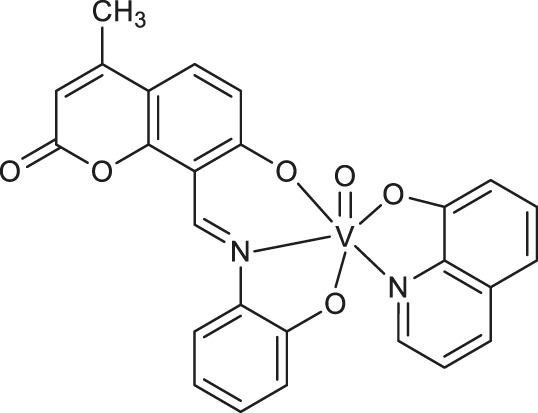	**50**	halo-peroxidase mimicking properties	Majumder and Rajak. (2020)
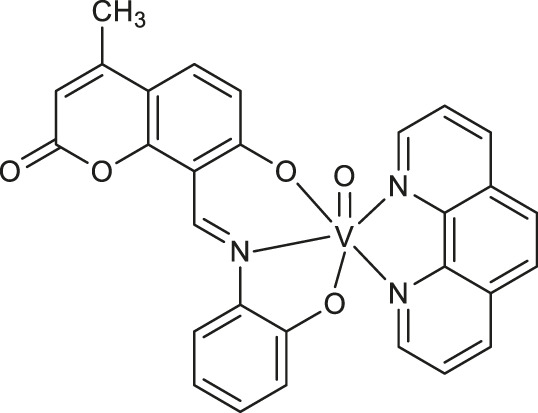	**51**	halo-peroxidase mimicking properties	Majumder and Rajak. (2020)
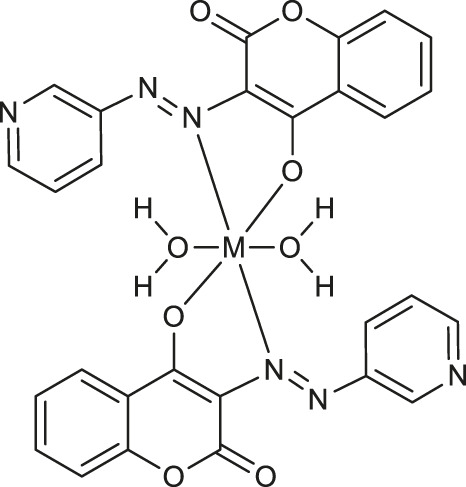	**52**: M = Cu	catecholase-like or phenoxazinone synthase mimicking properties	Sezgin et al. (2021)
	**53**: M = Mn		
**Enzyme inhibiting complexes**			
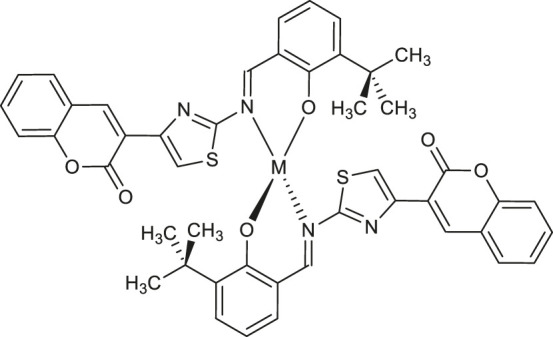	**54**: M = Pd	inhibition of acetyl-cholinesterase (AChE), butyryl-cholinesterase (BChE), and pancreatic cholesterol esterase (cease)	Şahin et al. (2020)
	**55**: M = Pt		
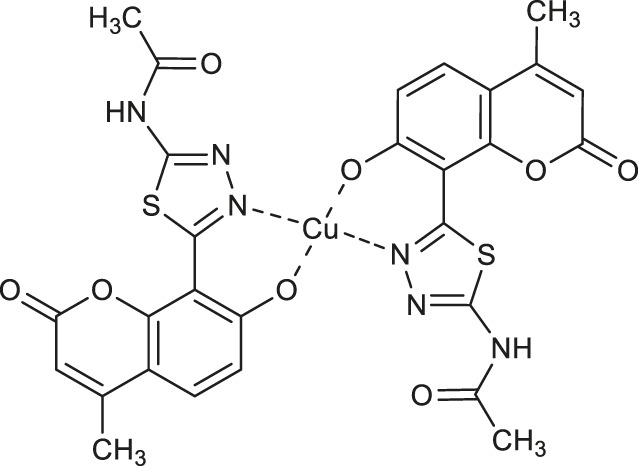	**56**	inhibition of acetyl-cholinesterase (AChE)	Karcz et al. (2021)
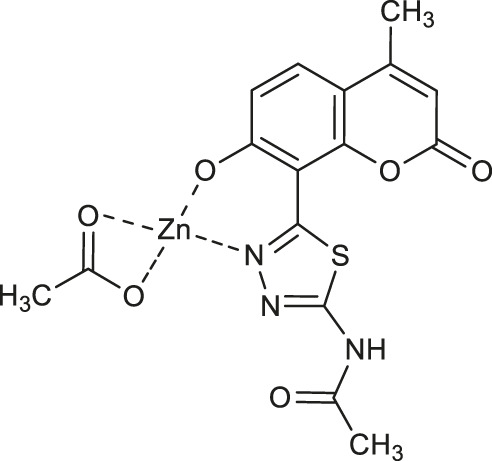	**57**	inhibition of acetyl-cholinesterase (AChE)	Karcz et al. (2021)

In the same year Gramni et al. designed and synthesized a new octahedral paramagnetic ruthenium (III) complex **5** of 4-{[*bis*(pyridin-2-ylmethyl)amino]methyl}-7-methoxy-2*H*-chromen-2-one (*chrdpa*) (Table 1), whose structure was proved by X-ray crystallography and TOF-mass spectrometry. Compound **5** exhibited *in vitro* cytotoxic effect against cervical cancer cells (HeLa) with an estimated IC_50_ of 137 μM. (Gramni et al., 2019). DNA binding studies performed by titrating calf-thymus DNA with **5** revealed a distinctive hypochromic effect by 33% in the UV-Vis spectrum profile indicating that the ruthenium (III) complex **5** is a groove binding agent.

It is noteworthy that such chemical species as copper and cobalt are also valuable for the development of antitumor compounds. In 2020, Lu W. et al. disclosed the synthesis of novel coumarin-metal complexes which may be useful in the treatment of cervical cancer (Lu et al., 2020). The reaction of 8-(*tert*-butyl)-3-(pyridin-2-yl)-2*H*-chromen-2-one with copper (II) nitrate trihydrate furnished the octahedral copper (II) complex **6** (Table 1) under mild reaction conditions, whose structure was unequivocally confirmed by a single-crystal X-ray diffraction analysis. This compound exhibited pronounced antiproliferative activity against the cervix tumor cell line (HeLa) with the IC_50_ value of 18.05 μM. Moreover, DNA binding studies revealed a hypochromic effect after adding DNA to the solution of complex 6, suggesting that this compound possesses an interactive mode of action.

Remarkable examples of novel cobalt (II) complexes have been presented by Mestizo and collaborators (Mestizo et al., 2021). Complexes **7** and **8** (Table 1) were synthesized starting from two tetradentate ligands bearing coumarin Schiff base (R = H, OCH_3_) and further evaluated against human cervical epithelioid carcinoma cell line (HeLa) and non-carcinogenic human cell lines (HFF-1 and HaCaT). The results of the *in vitro* study showed that cytotoxicity of **7** and **8** against HeLa cell line is higher than that of cisplatin with calculated IC_50_ values of 3.5 and 4.1 μM, respectively. Concerning the mechanism of action, it was explained that their anticancer potential on HeLa cells may be a result of the generation of reactive oxygen species (ROS).

Worth noting are cobalt (III) complexes [Co(B)_2_(L)]ClO_4_ **9-11** (Table 1) prepared by Sakar and co-workers in a two-step procedure, starting from *N*,*N*-donor bases: 1,10-phenanthroline (*phen*), dipyrido [3,2-days:2′,3′-*f*]quinoxaline (*dpq*) or dipyrido [3,2-*a*:2′,3′-*c*]phenazine base (*dppz*) and naturally occurring esculetin (6,7-dihydroxycoumarin) as dianionic *O*,*O*-donor, as well as cobalt (II) chloride and sodium perchlorate in the presence of triethylamine (Sarkar et al., 2021). These new complexes, due to their high visible light-triggered cytotoxicity against cervical cancer cell line (HeLa) and human breast cancer cell line (MCF-7), represent an interesting example of potent next-generation photochemotherapeutics. Moreover, complex **11** exerted a remarkable activity and negligible toxic effect in the dark, and binds with the greatest affinity to calf-thymus DNA. It was concluded that the observed photocleave of supercoiled DNA properties of this compound is connected with the formation of hydroxyl radicals from superoxide radicals *via* a type 1 photoredox reaction.

## Antimicrobial Complexes

With the rise of resistance to commonly used antibiotics, there is a need for novel therapeutic agents and drugs. It should be noted that therapeutic options for many bacteria strains are still limited and the research aimed at discovering new antimicrobial drugs has been focused on the design of metal complexes. Valuable information regarding antimicrobial metal complexes of coumarins can be found in recently published papers. These compounds are predicted to synergize with conventional antimicrobial drugs by binding to DNA or inhibiting DNA replication and biosynthesis (Claude et al., 2020; Frei., 2020).

In 2019, Belkhir-Talbi et al. described copper (II) and zinc(II) complexes of 3-(2-hydroxybenzoyl)-2*H*-chromen-2-one **12** and **13** (Table 1) as effective agents against Gram-positive bacteria strain: *Staphylococcus aureus* (ATCC 25923) (Belkhir-Talbi et al., 2019). It was found that both complexes exhibited moderate antibacterial activity and diameters of inhibition zones were in the range of 14–17 mm in comparison to reference second-generation cephamycin group antibiotic - cefoxitin (diameter of inhibition zone: 20 mm). Furthermore, complexes **12** and **13** showed a higher scavenging activity in comparison to the free ligand. The favorable *in silico* ADMET and drug-likeness profile of these compounds confirmed their non-toxic and non-carcinogenic properties.

Avdović et al. designed a series of five palladium (II) complexes with 3-[1-(2-hydroxypropylamino)ethylidene]chroman-2,4-dione, 3-[1-(phenylamino)ethylidene]chroman-2,4-dione, 3-[1-(*o*-toluidino)ethylidene]chroman-2,4-dione, 3-[1-(*m*-toluidino)ethylidene]-chroman-2,4-dione, and 3-[1-(2-mercaptoethylamino)ethylidene]chroman-2,4-dione in order to evaluate their antimicrobial activity (Avdović et al., 2019). In general, the activity of the complexes was higher or similar to that of the corresponding ligands. Among them, Pd(II) complex **14** (Table 1) was found to be more active against *Bifidobacterium animalis subsp. lactis*, *Pseudomonas aeruginosa*, *Escherichia coli* than the reference ligand - 3-[1-(2-hydroxypropylamino)ethylidene]chroman-2,4-dione and other complexes, showing favourable MIC and MMC values (500 and 1,000 μg/ml) towards probiotic bacteria - *Lactobacillus plantarum*. Moreover, complex **14** resulted to be more potent as fungicide compared with fluconazole, exhibiting a selective activity against *Aspergillus flavus* ATCC 9170 (MIC = 62.5 μg/ml). It was suggested that the promising antifungal activity of the complex **14** may be attributed to the presence of palladium (II) as well as hydroxypropylimine moiety.

In the same year, Almeida and collaborators synthesized a series of ruthenium (II)-DMSO complexes containing coumarin-*N*-acylhydrazone hybrids **15-18** (Table 1), whose antibacterial properties were evaluated (De Almeida et al., 2019). Investigation of biological activity revealed that the antibacterial potency of these compounds is higher than the free ligands and related to the lipophilicity (as indicated by the presence of chlorine and bromine atoms in substitution pattern), and charge of the complex. Among them, the *trans*-dichloro-*cis*-bis(dimethylsulfoxide)-(*Z*)-*N*′-4-bromobenzylidene-7-(diethylamino)-2-oxo-2*H*-chromene-3-carbhydrazide ruthenium (II) **17** (Table 1) turned out to be the most promising with MIC value of 31.24 μg/ml against *Staphylococcus aureus*. Surprisingly, further studies against tumor cell lines demonstrated that the potency of free ligands upon chelation was decreased.

In 2020, Yernale et al. evaluated the antimicrobial effects of a series of octahedral Co(II) **19**, Ni(II) **20**, Cu(II) **21**, and Zn(II) **22** (Table 1) complexes with coumarin-derived Schiff base ligand 2-[(7-hydroxy-4-methyl-2-oxo-2*H*-chromen-8-yl)methylene]-*N*-(4-phenylthiazol-2-yl)hydrazinecarboxamide against bacteria strains: *Bacillus subtilis*, *Staphylococcus aureus*, *Escherichia coli*, *Salmonella typhi* and prominent fungal pathogens: *Candida albicans*, *Aspergillus flavus*, *Cladosporium oxysporum*, *Aspergillus niger* (Yernale et al., 2020). According to the chelation theory, upon coordination of the metal ion to the O^N^O donor atoms Schiff base, all synthesized complexes were more active than the corresponding ligand as a result of the lipophilic character of chelate. This may promote the permeation of complexes through the membranes, blocking binding sites of the bacterial enzymes. Furthermore, the results of DNA cleavage studies showed that these newly synthesized complexes possess the ability to carry out an efficient cleavage of the supercoiled plasmid DNA pBR322, which suggests that these compounds constitute promising pathogenic microorganism inhibitors.

Most recently, according to the Nongpiur and collaborators report a half-sandwich complexes of platinum group metals were invented as antibacterial agents (Nongpiur et al., 2021). The complexes of Ir(III), Ru(II), and Rh(III) with coumarin-*N*-acylhydrazone-based ligands **23-26** (Table 1) have shown promising activities towards Gram-positive bacterial strains: *Staphylococcus aureus* and *Bacillus thuringiensis* with MIC in the range of 1.25−2.5 mg/ml and the inhibition zones in the range of 18-19 mm. The presence of the electron-donating groups N(C_2_H_5_)_2_ or OCH_3_ in the structure of these complexes might be contributed to increased antibacterial activity. Additionally, the evaluation of redox properties of these compounds showed their moderate to high radical scavenging activity (50.6−85%). In this context, described platinum group metal complexes of coumarins are also worth mentioning as potential antioxidants which can neutralize the free radicals and inhibit the propagation of the chain reactions.

With regards to the antimicrobial activity of coumarin-based complexes some studies have been recently carried out showing the potential of palladium (II) complexes as antiviral agents. Milenković and co-workers have studied *in silico* a series of palladium (II) complexes **27−29** (Table 1) as potent inhibitors of the main protease (M^pro^) of severe acute respiratory beta-coronavirus (SARS-CoV-2) (Milenković et al., 2020). The computational studies showed that the binding affinities of complexes **27−29** were higher (*K*_i_ = 2.33; 7.55; 17.94 nM) than those obtained for two potential SARS-CoV-2 M^pro^ inhibitors - cinanserin and chloroquine (*K*_i_ = 256.8 nM, and 629.1 nM, respectively). Moreover, the generated binding free energy of complex **28** is significantly high (Δ*G*_binding_ = −149.7 kJ/mol) compared to the values of the free ligand (Δ*G*_binding_ = −62.1 kJ/mol) and reference cinanerin (Δ*G*_binding_ = −49.8 kJ/mol). Based on the promising molecular docking results, it was claimed that further experimental studies should be undertaken to verify these predictions.

## Antioxidant Complexes

The antioxidant potential of coumarin-metal complexes has been investigated in recent years and it was presented that they have a special ability to scavenge reactive oxygen species in biological systems. The uncontrolled production of reactive oxygen species (ROS) is associated with many pathologies such as tumors, neurodegenerative diseases, sickle cell anaemia, or thalassemia. However, these disorders may be treated using compounds with free radicals scavenging properties, which at the same time possess the ability to protect the normal blood cells. In this context, the hematologic cancers therapeutic strategy requires antioxidants combating the tumor cells and exhibiting high compatibility with erythrocytes. Additionally, some of them possess potential anti-haemolytic properties.

In 2020, Geetha and others evaluated three series of silver(I) complexes of coumarin and allyl substituted NHC ligands derived from 1,2,4-triazole for their antioxidant properties (Geetha and Narayana., 2020). However, all the complexes displayed much better DPPH-based radical scavenging activity compared with silver nitrate and a representative triazolium hexafluorophosphate salt, bis-NHC coordinated silver hexafluorophosphate complexes **30–32** (Table 1) evidenced higher activity than other members of the tested complexes. The best result was found for complex **30** with an IC_50_ value of 61 μM. In addition, at a concentration of 100 μM complexes **30–32** exhibited lysis of red blood cells as low as 2.32%. Efforts to obtain even more potent bioactive agents have led to new Ag(I)-NHC complexes derived from coumarin substituted 4-aryl-1,2,4-triazol-5-ylidenes **33** and **34** (Table 1) which displayed antioxidant potencies with the IC_50_ values of 7.49 and 7.42 μM, respectively, and at the concentration of 100 μM showed cell lysis of human red blood cells in the range of 3.50–4.38% (Geetha and Małecki., 2020).

Recently, it was demonstrated, that chemotherapeutic properties of coumarin-3-carboxylic acid (2-oxo-2*H*-1-benzopyran-3-carboxylic acid, HCCA) may be increased by metal chelation. One example is the coordination of HCCA with a silver(I), which was proved to enhance its antimicrobial activity. Such promising biological activity of HCCA and its complexes prompted their further investigations. For this reason, the antioxidant activity of transition metal complexes of HCCA has been screened *via* the 2,2-diphenyl-1-picrylhydrazyl (DPPH) method by De Souza and co-workers (Souza et al., 2021). The best radical scavenging properties have been shown by copper (II) complex **35** (Table 1), which exhibited 31% of inhibition of free radicals at a concentration of 160 µM.

Another example of promising antioxidants represents synthesized in 2020 by Özdemir et al. novel 7-oxy-3-ethyl-6-hexyl-4-methylcoumarin substituted lutetium (III) phthalocyanine compounds (Özdemir and Kӧksoy., 2020). The radical cation scavenging activities of complexes **36** and **37** (120.344 mM trolox/mg and 188.733 mM trolox/mg, respectively) (Table 1) were significantly higher than the values for standard BHA - butylated hydroxyanisole (52.63 mM trolox/mg) in 2,2′-azino-bis-3-ethylbenzothiazoline-6-sulfonic acid (ABTS) analysis.

## Complexes as Photosensitizers in Photodynamic Therapy

Photodynamic therapy (PDT), an important strategy for anticancer treatment, involves specific compounds called photosensitizers, oxygen, and light irradiation at an appropriate wavelength to induce the production of high levels of singlet oxygen by photosensitizer. Effective photosensitizers should also exhibit required stability to not decay after light exposure in photodegradation phenomenon. The increase of singlet oxygen or other reactive oxygen species may promote oxidative stress and apoptosis of cancer cells with low ROS levels (Kim et al., 2013; Qian et al., 2018).

In 2020, Özdemir and co-workers exploited the copper(I)-catalyzed azide-alkyne Huisgen 1,3-dipolar cycloaddition reaction to obtain the first example of phthalocyanine-coumarin derivatives bearing triazole ring, which constitute the direct precursors of peripheral **38–39** or non-peripheral **40–41** zinc(II) and magnesium (II) phthalocyanines (Zn-Pcs or Mg-Pcs) (Table 1) (Özdemir and Kӧksoy., 2020). The photophysical and photochemical studies revealed that complexes **38–41** may be used as type II photosensitizers in photodynamic therapy because the singlet oxygen quantum yields (Φ_Δ_) ranged in acceptable values from 0.17 to 0.49; the highest Φ_Δ_ value was observed for the peripheral zinc phthalocyanine **38** (Φ_Δ_ = 0.489 in DMSO). Moreover, magnesium-Pc **39** showed an acceptable value of the fluorescence quantum yield Φ_F_ = 0.31. At this point it should be mentioned, that Φ_Δ_ values of metal-free coumarin/triazole phthalocyanine compounds were obtained as quite low.

The encouraging results in the perspective of using metal complexes of highly water-soluble quaternized ionic phthalocyanines **42-44** and **45-47** (Table 1) as promising photosensitizers for photodynamic therapy were obtained by Boyar and Çamur (Boyar and Çamur. 2019). The highest singlet oxygen quantum yields in the presence of Triton X-100 exhibited peripherally substituted In(III)Pc **43** (Φ_Δ_ = 0.93) and non-peripherally substituted - Zn(II)Pc **45** (Φ_Δ_ = 0.92), and In(III)Pc (Φ_Δ_ = 0.41). Moreover, complexes of Pcs **42-44** and **45-47** readily bind to bovine serum albumin, which suggests that they will be carried in the bloodstream similar to drugs.

Commonly used chemotherapeutics in the treatment of an advanced prostate cancer exhibit several disadvantages and possess limited efficiency to target cancer stem cells (CSCs). The CSCs are considered the main reason for tumor metastasis and antitumor drugs resistance. Moreover, these cancer stem cells characterize the low levels of ROS. In this context, the strategy of stimulation of production of several types of ROS may be beneficial in the treatment of aggressive and even hardly treatable cancer stem cells. Novohradsky and co-workers have described a cyclometalated iridium (III) complex **48** conjugated with novel class of far-red/NIR-emitting fluorophore (COUPY), in which carbonyl group of coumarin core was replaced with *N*-alkylated cyano (4-pyridine)methylene moiety to enhance the push-pull character of aromatic ring (Table 1) (Novohradsky and Rovira., 2019). The Ir(III)-COUPY conjugate **48** was proven to be very promising photosensitizer suitable for photodynamic therapy (PDT) of cancers, including hypoxic tumors. For the first time it was demonstrated that such photo-induced therapy with metal-based compound **48** might provide a new approach for prostate cancer treatment due to the controlled cytotoxic effect by generation a specific type I ROS, superoxide anion radical, upon visible-light irradiation at a dose of 28 J cm^−2^ of 420 nm blue light (Novohradsky and Markova et al., 2021). The pronounced selectivity for tumor cells compared with non-cancerous cells, as well as low toxicity in prostate cancer cell line DU145 in the dark (IC_50 (irrad.)_ = 5.7 μM *vs* IC_50 (dark)_ ≥ 100 μM) could results in low side effects and reduced damage of normal cells during the PDT.

## Enzyme-Mimicking Complexes

Due to the fact, that metal ions play a pivotal role in biological systems as centers of enzymes or metalloenzymes, transient metal complexes of small molecules may constitute a valid tool in the field of biochemistry and modern biosynthesis as enzyme mimetics.

The halo-peroxidase mimicking properties of oxidovanadium complexes **49-51** (Table 1) have been proved by Majumder et al. (Majumder and Rajak., 2020). The complexes were studied on a model catalytic bromination reaction of aromatic aldehydes. In this case, the oxidative conversions of aldehyde in the presence of hydrogen peroxide and potassium bromide in an aqueous medium were very effective.

In 2021, Sezgin and co-workers exploited two complexes [Cu(PYC)_2_(H_2_O)_2_] **52** and [Mn(PYC)_2_(H_2_O)_2_] **53** (Table 1) containing copper (II) and manganese (II) as catecholase-like or phenoxazinone synthase mimetics under aerobic conditions (Sezgin et al., 2021). The kinetic studies of enzymatic activity revealed that copper (II) complex was more effective than manganese (II) one and both complexes possess better catecholase-like activity than phenoxazinone synthase mimetic properties, These results indicate that complexes 52 and 53 represent a good starting point for the design of promising tools for the catecholase-like and phenoxazinone synthase-like activities.

## Enzyme Inhibiting Complexes

Şahin et al. demonstrated the inhibitory potency of palladium (II) and platinum (II) complexes of coumarin-based ligand - 3-{2-[(3-(*tert*-butyl)-2-hydroxybenzylidene)amino]thiazol-4-yl}-2*H*-chromen-2-one **54** and **55** (Table 1) against acetylcholinesterase (AChE), butyrylcholinesterase (BChE), and pancreatic cholesterol esterase (cease) (Şahin et al., 2020). The inhibitory properties against AChE, BChE, and cease of synthesized complexes were determined by a standard procedure involving the spectrophotometric measurements according to Elman or Pietsch and Gütschow methods. It was found that **54** exhibited a strong inhibition towards acetylcholinesterase (AChE) with IC_50_ value of 16 μM, compared to the pyridostigmine bromide as a positive control (IC_50_ = 23 μM), whereas platinum complex **55** showed inhibitory activity on three esterases with following IC_50_ values: 12 μM (AChE), 23 μM (BChE), and 21 μM (cease). These results supported by Density Functional Theory (DFT) calculations are encouraging in the perspective of using coumarin-metal complexes with dual cholinesterase inhibition (AChE and BChE) as potential anti-Alzheimer agents.

Concerning the anti-AChE properties of coumarins, the structure-activity studies pointed out that the presence of an amide moiety in their structure may play important role in the enzyme inhibition (Asadipour et al., 2013; Ghanei-Nasab et al., 2016).

Karcz and collaborators evaluated a series of copper (II) and zinc(II) complexes of novel coumarin-thiadiazole hybrids as potential acetylcholinesterase inhibitors (Karcz et al., 2021). In this assay it was identified that the most active are the amide-bearing complexes **56** (IC_50_ = 0.174 μM) and **57** (IC_50_ = 0.184 μM), as well as the parent ligand - *N*-[5-(7-hydroxy-4-methyl-2-oxo-2*H*-chromen-8-yl)-1,3,4-thiadiazol-2-yl]acetamide (IC_50_ = 0.181 μM). Despite the fact, that these hybrids exhibited about 4-fold weaker activities compared to that of the commercially available AChE inhibitor - tacrine (IC_50_ = 0.053 μM), the result obtained set the direction for potential further modifications of this type of compounds in order to obtain their amide derivatives with higher anti-neurodegenerative potency. The observed significant decrease in solubility of metal complexes in relation to their free ligands suggests that future studies should also focus on their pharmacokinetic properties. In addition, since the AChE is not the only target in the treatment of the neurodegenerative process, new approaches ought to aim at a more detailed evaluation of the anti-neurodegenerative properties of these complexes, for example, the assessment of butyrylcholinesterase (BuChE) inhibiting activity, as well as amyloid fibrils suppression ability.

## Coumarin-Based Metal Complexes as Fluorescent Probes/Sensors

Recently, the fluorescent methods of detection or monitoring of a variety of biologically important species such as biothiols or ions under physiological conditions in intracellular compartments (cell nucleus, mitochondria, and cytosol) have been developed. In this context the metal complexes of coumarins, which represent the electron-rich conjugated systems with strong fluorescent properties, have found important applications also in the selective detection of biomaterials, metals and ions.

Given the fact that altered cellular glutathione (GSH) levels are related to human diseases such as cancer, AIDS, cardiovascular or neurodegenerative disorders, in 2019 He and others proposed a fluorescent probe for glutathione based on a copper complex of coumarin hydrazide Schiff base derivative (He et al., 2019). The fluorescent Schiff base ligand - *N*'-[(1*H*-imidazol-2-yl)methylene]-7-(diethylamino)-2-oxo-2*H*-chromene-3-carbohydrazide was reacted with copper (II) perchlorate (Cu(ClO_4_)_2_·6H_2_O) yielding an appropriate copper (II) complex **58** (Table 2). First, it was observed that fluorescence quenching of ligand was induced by copper (II) ions and the fluorescence quenching effect was not disturbed by the presence of other metal ions. The mechanism of fluorescence quenching may be attributed to the photoinduced charge transfer (PCT). Then, the complex **58** was evaluated as a “turn-on” fluorescent probe for optical detection of glutathione (GSH). The test pointed out, that the stronger ability of GSH to capture copper (II) ions from complex **58** results in its demetallation and subsequent fluorescence restoration. The use of complex **58** gives the minimum detection limit of GSH about 0.12 μM.

**TABLE 2:** Coumarin-based metal complexes as fluorescent probes/sensors.

Chemical structure	Number of complex	Application	References
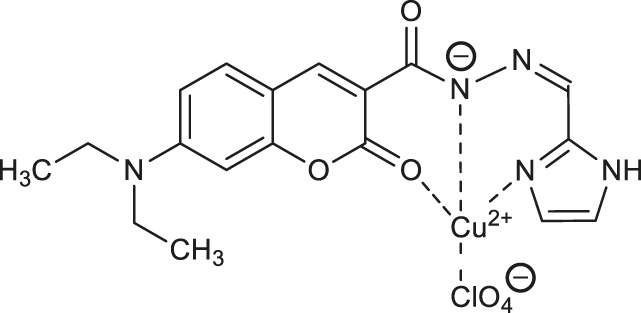	**58**	detection of glutathione (GSH)	He et al. (2019)
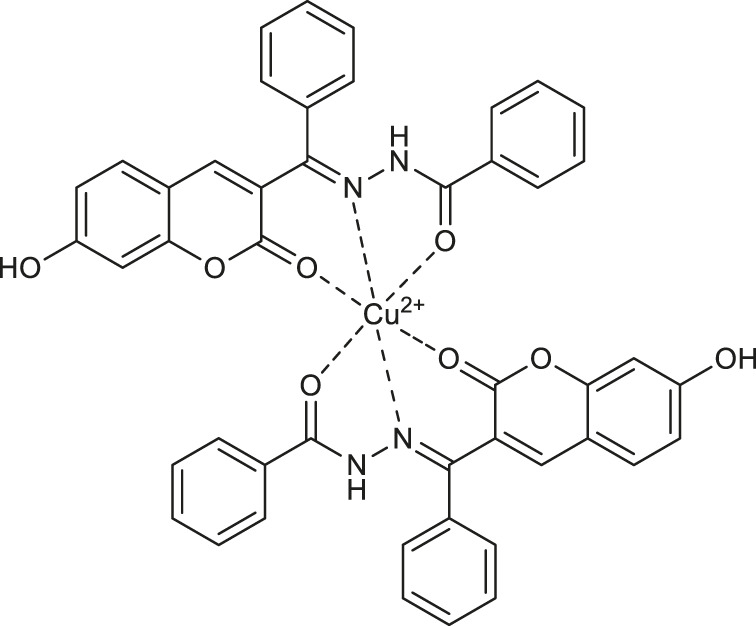	**59**	detection of cysteine (Cys), homocysteine (Hcy), and glutathione (GSH)	Li et al. (2019)
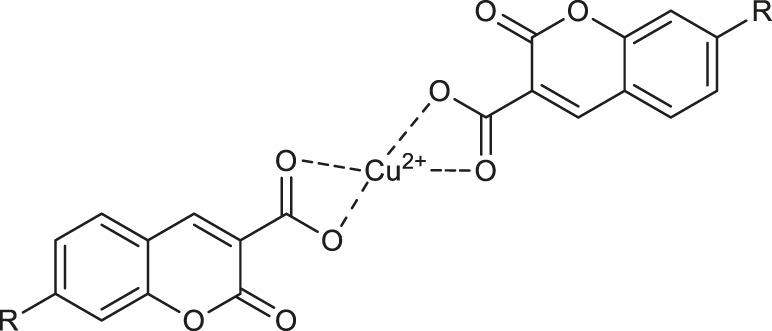	**60**: R = OH	detection of water	Cheng et al. (2019)
	**61**: R = N(C_2_H_5_)_2_		
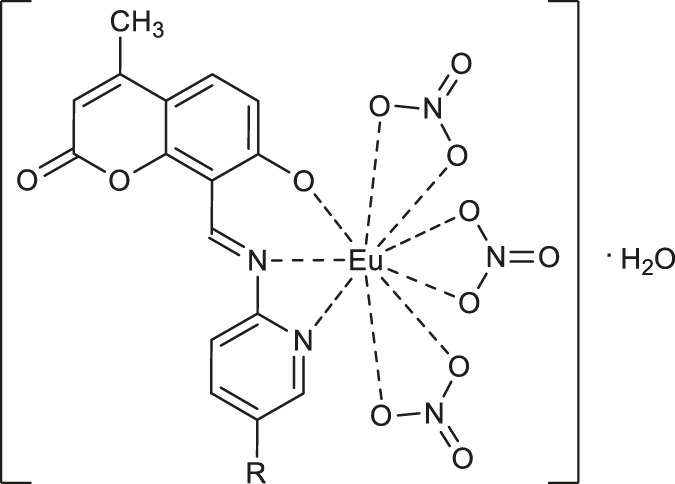	**62**: R = H	detection of fluoride anion (complex **63**)	Luan et al. (2020)
	**63**: R = OCH_3_		
	**64**: R = NO_2_		
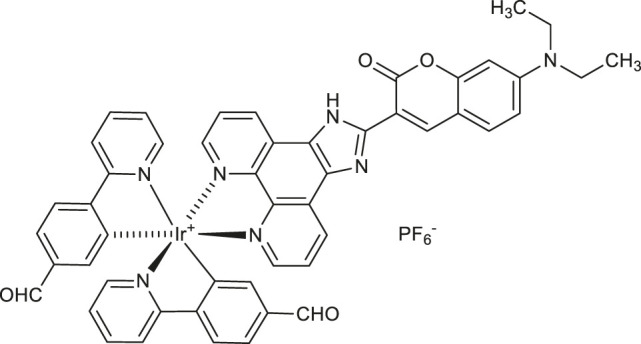	**65**	detection of thrombin	Li et al. (2020)
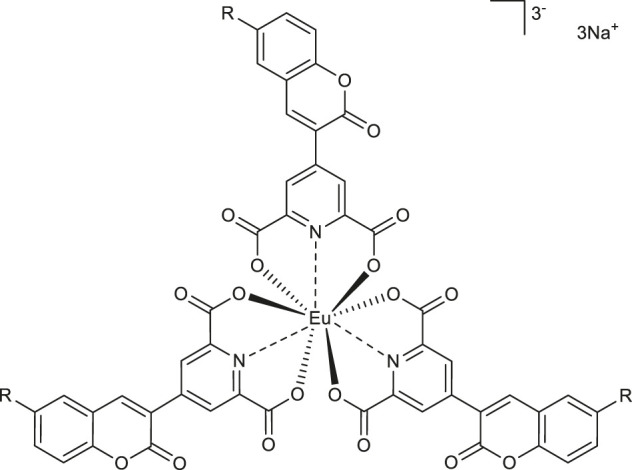	**66**: R = H	constructing of molecular probes	Di Pietro et al. (2021)
	**67**: R = I		

In 2019, Li et al. reported formed “*in situ*” in acetonitrile and water mixture coumarin-based copper (II) complex **59** (Table 2) that may find application as a fluorescent turn-off chemosensor for colorimetric and fluorescent detection of biothiols: cysteine (Cys), homocysteine (Hcy), and glutathione (GSH) (Li et al., 2019). The high affinity of thiols to the Cu^2+^ results in the demetallation of the Cu-complex **59**. In turn, releasing of free ligand turn off the fluorescence, in contrast, to “turn-on” chemosensors for biothiols based on the demetallation of copper-complexes. The estimated detection limits for Cys, Hcy, and GSH were in the range of 10-16 μM.

Concerning the fluorescent properties of copper (II) complexes with coumarin-based ligands, Cheng and collaborators developed an efficient fluorescent “turn-on” water sensors (Cheng et al., 2019). It was demonstrated that 7-R-2-oxo-2*H*-chromene-3-carboxylic acids (R = OH, N(C_2_H_5_)_2_) in dry organic solvents generate “*in situ*” almost non-fluorescent copper (II) complexes **60** and **61** (Table 2), whereas in the presence of a trace of water complexes **60** and **61** exhibit bright emission.

In 2020, Luan and co-workers reported the synthesis of europium (III) complexes [EuL (NO_3_)_3_·H_2_O] **62-64** (Table 2) based on the novel coumarin Schiff base derivatives, which exhibited characteristic red fluorescence and showed the influence of different substituents on the fluorescence properties of these complexes in order OCH_3_>H > NO_2_. (Luan et al., 2020). Furthermore, it was demonstrated, that the characteristic red fluorescence intensity of complex **63** was significantly reduced in the presence of fluoride anion. The response limit in the range of 1.0 · 10^−5^–2.2 · 10^-5^ mol L^−1^ clearly indicates that fluorescent europium (III) complex **63** may serve as a selective tool for fluoride anion detection.

Recently, photoelectrochemical (PEC) techniques based on metal complexes have been applied in the detection of diverse biological analytes. Coumarin-attached cyclometalated iridium (III) complex [(CHO-bpy)_2_Ir(C-phen)]^+^PF_6_^−^ **65** (Table 2) synthesized by Li et al. was proved as efficient PEC active material suitable in bioanalysis due to the enhanced absorption in the visible light (Li et al., 2020). PEC sensor **65** exhibited enhanced fine selectivity and sensitivity for thrombin with the detection limit of 23 fM.

Finally, Di Pietro et al. synthesized coumarin-based dipicolinate europium complexes Na_3_ [Eu (L_1_)_3_] **66** and Na_3_ [Eu (L_2_)_3_] **67** (Table 2) (Di Pietro et al., 2021), which as either mono or dual emitter merge the excellent photophysical properties of the 2-oxo-2*H*-chromene chromophore and europium ion. Their simple synthesis and possible chemical modification give the opportunity to optimize their emission properties in constructing molecular probes.

## Conclusion

In recent years, the considerable increase in the number of papers and reports describing the possible uses of coumarin-based metal complexes in therapy and medicine is a vivid demonstration of their impact on the scientific community. Coumarin-based complexes have also attracted attention due to their interesting fluorescent properties as sensors or probes.

In this mini-review we summarized the recent advances in coumarin-metal complexes as anticancer, antimicrobial, antifungal, or antioxidants agents as well as enzyme mimics or enzyme inhibitors from 2019 to 2021.

Having in mind a great need for new therapeutic agents for combating cancers, metal complexes with coumarin-based ligands have been extensively studied for their cytotoxic properties. The most promising coumarin-based compounds incorporating metal ions are bi-functional platinum (IV) complexes with COX inhibiting properties or Ir(III)-COUPY conjugates as promising photosensitizers in photodynamic therapy targeting cancer stem cells.

On the other hand, with the rise in the number of multi-drug resistant microbial pathogens, the development of new antimicrobial agents is another main research in which coumarin-based metal complexes are evaluated.

The valuable and promising compounds incorporating coumarin-metal complexes are also those which may serve as lead compounds for developing anti-neurogenerative agents.

Moreover, the most recent advances on the synthesis of metal complexes with coumarin-based ligands include probes or sensors with practical applications for detecting diverse biological analytes under physiological conditions. Great interest has been made towards the development of coumarin-based copper (II), iridium (III) or europium (III) complexes as fluorescent probes or PEC sensors for bioanalysis.

Summing up, the reported studies and some others outside the scope of this mini-review (i.e. light-emitting diodes (Feng et al., 2019; Feng et al., 2020; Yang et al., 2021), catalysts (Dharani et al., 2019; Akpunar et al., 2021; Gautam et al., 2021; Karataş et al., 2021) evident the great potential of coumarin-metal complexes in different fields of research. We hope that this mini-review will be useful in the further development of coumarin-based metal complexes in drug design and fluorescence probes.
